# Modeling of Cerebral Oxygen Transport Based on *In vivo* Microscopic Imaging of Microvascular Network Structure, Blood Flow, and Oxygenation

**DOI:** 10.3389/fncom.2016.00082

**Published:** 2016-08-31

**Authors:** Louis Gagnon, Amy F. Smith, David A. Boas, Anna Devor, Timothy W. Secomb, Sava Sakadžić

**Affiliations:** ^1^Optics Division, Department of Radiology, MHG/MIT/HMS Athinoula A. Martinos Center for Biomedical Imaging, Massachusetts General Hospital and Harvard Medical SchoolCharlestown, MA, USA; ^2^Institut de Mécanique des Fluides de ToulouseToulouse, France; ^3^Department of Physiology, University of ArizonaTucson, AZ, USA; ^4^Departments of Neurosciences and Radiology, University of California, San DiegoLa Jolla, CA, USA

**Keywords:** cerebral blood flow (CBF), cerebral blood flow measurement, cerebrovascular circulation, brain imaging methods, modeling and simulations

## Abstract

Oxygen is delivered to brain tissue by a dense network of microvessels, which actively control cerebral blood flow (CBF) through vasodilation and contraction in response to changing levels of neural activity. Understanding these network-level processes is immediately relevant for (1) interpretation of functional Magnetic Resonance Imaging (fMRI) signals, and (2) investigation of neurological diseases in which a deterioration of neurovascular and neuro-metabolic physiology contributes to motor and cognitive decline. Experimental data on the structure, flow and oxygen levels of microvascular networks are needed, together with theoretical methods to integrate this information and predict physiologically relevant properties that are not directly measurable. Recent progress in optical imaging technologies for high-resolution *in vivo* measurement of the cerebral microvascular architecture, blood flow, and oxygenation enables construction of detailed computational models of cerebral hemodynamics and oxygen transport based on realistic three-dimensional microvascular networks. In this article, we review state-of-the-art optical microscopy technologies for quantitative *in vivo* imaging of cerebral microvascular structure, blood flow and oxygenation, and theoretical methods that utilize such data to generate spatially resolved models for blood flow and oxygen transport. These “bottom-up” models are essential for the understanding of the processes governing brain oxygenation in normal and disease states and for eventual translation of the lessons learned from animal studies to humans.

## Introduction

The energy requirements of the cerebral cortex are met almost exclusively by oxidative metabolism of glucose, necessitating continuous delivery of oxygen by diffusion into the tissue from flowing blood (Buxton and Frank, 1997; Attwell and Laughlin, 2001; Raichle and Gusnard, 2002; Lin et al., 2010; Hall et al., 2012). The distance that oxygen can diffuse from blood into oxygen-consuming tissue is limited. An approximate upper bound is the one-dimensional diffusion distance, *L*_*D*_ = (2*D*α*P*_0_/*M*)^1/2^, where *D* and α are the diffusivity and solubility of oxygen in tissue, *P*_0_ is the partial pressure of oxygen (pO_2_) in blood, and *M* is the rate of oxygen consumption (Secomb et al., 1993). Typical values for rodent cerebral cortex are *D*α = 6 × 10^−10^ cm^3^O_2_/cm/s/mmHg, *P*_0_ = 50 mmHg and *M* = 6 cm^3^O_2_/100 g/min, resulting in *L*_*D*_ ≈ 77 μm (Secomb et al., 2000). The tissue must therefore be supplied by a dense network of microvessels (Hirsch et al., 2012; Blinder et al., 2013), so that each point in the tissue is within a distance *L*_*D*_ of the nearest vessel. A further implication of this result is that significant gradients in PO_2_, over a scale of tens of μm, are present in the tissue surrounding each microvessel that is contributing to the diffusive delivery of oxygen between blood and tissue (Popel, 1989; Pittman, 2005; Goldman, 2008). This does not necessarily imply that significant gradients in PO_2_ are present around all microvessels. For instance, if the oxygen demand of the tissue is largely met by diffusion from arterioles (Sakadžić et al., 2014), then PO_2_ gradients around neighboring capillaries are correspondingly reduced. This is more likely to occur at lower oxygen consumption rates (Secomb and Hsu, 1994). As a further example, heterogeneous levels of oxygen within capillaries can lead to situations where some capillaries do not participate in diffusive oxygen delivery and may even take up oxygen (Secomb and Hsu, 1994).

The oxygen demand of cerebral cortex depends on the level of neuronal activity, which varies in space and time across cortical regions. However, in spite of significant research efforts, our current understanding of the neurovascular coupling mechanisms by which blood flow and oxygen delivery are coordinated with spatially and temporally varying metabolic demands in the brain remains incomplete in both healthy and diseased brain (Iadecola, 2004; Girouard and Iadecola, 2006; Raichle and Mintun, 2006; Iadecola and Nedergaard, 2007; Gordon et al., 2008; Cauli and Hamel, 2010; Lecrux et al., 2011; Devor et al., 2012). In the context of task-induced hemodynamic activity in a healthy subject, neurovascular coupling is commonly associated with the causal chain of events, where changes in neural activity drive changes in energy metabolism which then drive changes in blood flow (Raichle and Mintun, 2006). However, recent experimental data suggests an alternative hypothesis that much of the acute vasodilation and constriction under healthy conditions appears to be driven by molecules related to neural signaling itself, i.e., release of vasoactive signaling agents such as neuropeptides, prostaglandins, nitric oxide and, possibly, K^+^ from active neurons (Attwell and Iadecola, 2002; Cauli and Hamel, 2010; Kleinfeld et al., 2011). This implies that CBF and CMRO_2_ are driven in parallel by neural activity, and potentially by different aspects of neural activity (Devor et al., 2012; Buxton et al., 2014).

**Figure 1:**
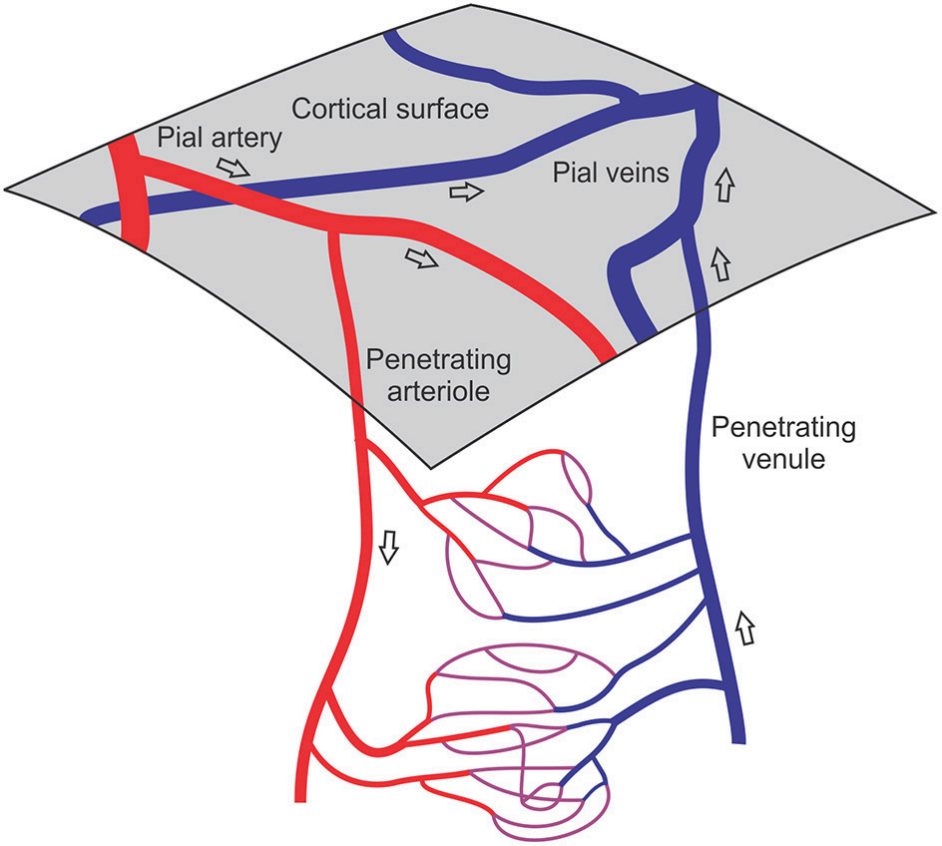
**Schematic illustration of cortical vasculature, showing network structures involved in neurovascular coupling**. The cerebral cortex receives its blood supply from a mesh of pial arteries and veins lying on the cortical surface. Penetrating arterioles and venules branch off the pial vessels and traverse the thickness of the cortex, supplying dense arrays of capillaries. Transient activation of neuronal signaling leads to an increased metabolic demand in a tissue region, necessitating increased blood flow, which is achieved by adjustment of vascular diameters in the upstream vessels feeding that region and possibly also in the capillaries and downstream vessels. Open arrows indicate direction of blood flow.

The mechanisms by which blood flow and oxygen delivery are coordinated with spatially and temporally varying metabolic demand in the brain critically depend on vascular network-level interactions. In the present review, we focus on the microvascular networks (Figure 1) where the blood flow rate in any given segment depends not only on the flow resistance of that segment but also on the distributions of resistance and blood flow in the vessel segments to which it is connected. An increase in flow in a given segment requires dilation of the upstream arterioles supplying that segment, in addition to possible dilation of the segment itself and downstream segments. Furthermore, the oxygen content of blood arriving at a given segment depends on the extraction that has already occurred, and is therefore affected by oxygen transport phenomena occurring along the upstream part of the flow pathway. Conducted responses, in which vasodilator or vasoconstrictor signals travel upstream from capillaries along arterioles, play an important role by coordinating upstream dilation (Jensen and Holstein-Rathlou, 2013).

A further challenge in understanding the dynamics of tissue oxygenation is the heterogeneity of structural and hemodynamic parameters in the microcirculation (Pries et al., 1995). The significance of heterogeneous blood flow, resulting in high capillary transit time heterogeneity (CTH), has been highlighted by Jespersen and Østergaard (2012). By introducing variation in capillary velocities in an array of capillaries, they showed that high CTH can lead to reduced oxygen extraction. A thorough analysis of the consequences of heterogeneity requires consideration of microvascular network structure.

The value of theoretical models for gaining quantitative understanding of oxygen transport to tissue has long been recognized (Krogh, 1919). A variety of approaches have been used. In the Krogh cylinder model (Krogh, 1919), the exchange of oxygen between a single capillary and a surrounding cylinder of tissue is analyzed, taking into account the radial gradients of oxygen concentration in the tissue. The application of this approach to cerebral oxygen transport is discussed by Mintun et al. (2001). Other approaches have been based on the assumption that oxygen efflux from capillaries is proportional to plasma oxygen concentration (Buxton and Frank, 1997) or proportional to the difference between intravascular and tissue oxygen level, where the tissue space is represented as a well-mixed compartment, characterized by a specific tissue oxygen level (Jespersen and Østergaard, 2012). In order to represent the contributions of different classes of vessels to oxygen transport, a compartmental model was developed by Sharan et al. (1989).

While these modeling approaches provide useful insights into aspects of cerebral oxygen transport, they do not explicitly represent the effects of network structure and flow distribution. Detailed network-level models are valuable because they permit analysis of the effects of microvascular network architecture and flow distribution on the distribution of oxygen in the tissue (Popel, 1989; Secomb et al., 2000, 2004; Goldman, 2008). Such models require extensive experimental data as input, including information on the three-dimensional structure of microvascular networks containing a large number (typically hundreds or thousands) of segments, together with at least partial information on vessel blood flow rates and oxygen content, and estimates of key oxygen transport parameters. Until recently, however, experimental data available for modeling were mostly from planar or restricted three-dimensional structures, flow rates were generally not measured in the same structures, and data on tissue oxygenation were rarely available.

Recently, high-spatial resolution optical imaging technologies capable of penetrating up to 1 mm into brain tissue have been developed and applied to *in vivo* brain imaging, providing enhanced capabilities to obtain the data needed for such models. The advantages of optical imaging include high temporal and spatial resolution within reasonable thicknesses of tissue, numerous methods for enhancing contrast, and modest cost of instrumentation. To provide the data needed for network-level modeling of microvascular blood flow and oxygen transport, the imaging modality should meet the following requirements. (i) It should allow imaging of depths of several hundred μm into the tissue, to enable three-dimensional mapping of microvascular structures spanning a substantial fraction of the cortical thickness. (ii) It should have high spatial resolution, sufficient to resolve individual capillaries. (iii) It should allow estimation of blood flow rates in individual microvessels. (iv) It should allow estimation of spatially resolved oxygen levels in microvessels and/or the surrounding tissue. (v) For studies of neurovascular coupling, it should have high temporal resolution (~1 s), sufficient to resolve the time course of hemodynamic changes. In this article, we review state-of-the-art optical microscopy technologies for quantitative *in vivo* imaging of cerebral microvascular structure, blood flow, and oxygenation. We then review strategies for network-level modeling of cerebral microcirculation using data obtained from such *in vivo* imaging. These methods will contribute to future investigations of neurovascular and neurometabolic coupling and provide the essential bridge for translation from the microscopic to the network/systems level needed for human translation.

## Optical imaging of cerebral microvascular structure, hemodynamics and oxygen transport

### Optical imaging of cerebral microvascular structure

Optical microscopy techniques that have been applied *in vivo* to image the cerebral microvasculature in rodents include multi-photon laser scanning microscopy (MPM), optical coherence tomography (OCT), and photoacoustic imaging (PAI). Other “methods to watch” are light-sheet (Keller and Ahrens, 2015) microscopy and super-resolution ultrasound (US) (Errico et al., 2015). The former has been initially targeted to relatively small and transparent organisms and tissue samples (Huisken et al., 2004) but was recently adapted for imaging of neurovascular activity in the mouse cerebral cortex *in vivo* albeit with limited depth penetration (Bouchard et al., 2015). The latter started as a slow imaging modality (Viessmann et al., 2013; Christensen-Jeffries et al., 2015), but the most recent report achieved mapping of the vasculature in the whole rat brain with ~8 μm resolution within tens of seconds (Errico et al., 2015).

**Figure 2:**
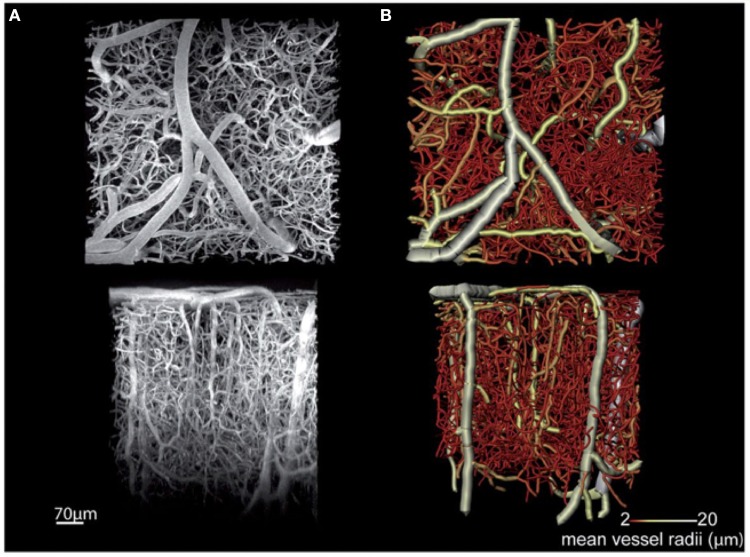
**MPM microangiography**. *In vivo* multi-photon fluorescence microscopy images of the cortical microvasculature in a 3-month old non-transgenic mouse. Acquired in the primary somatosensory cortex, this maximum intensity projection image **(A)** shows penetrating arterioles running vertically down from the cortical surface (top of the bottom row image) as well as ascending venules interspersed with a dense capillary network. **(B)** A tubular model obtained by segmentation of the branching vessel network shown in **(A)**. The color bar indicates the coding of average vessel diameters. *Top* row images are parallel with the cortical surface, while *bottom* row images are perpendicular to the cortical surface. Adapted with permission from Dorr et al. (2012).

Among these techniques, MPM is the most mature and widely applied for *in vivo* acquisition of high- resolution cortical angiograms (Helmchen and Denk, 2005; Svoboda and Yasuda, 2006). MPM detects photoluminescence (fluorescence or phosphorescence) from endogenous or exogenous chromophores, and images are typically created from the sequences of sequential point measurements. Brain imaging with MPM can be performed through the thinned skull in mice (Drew et al., 2010), although more commonly a glass-covered cranial window is used allowing increased penetration depth into the brain. Images with a high signal-to-noise ratio are routinely obtained down to a 600–700 μm cortical depth by commercially available MPM setups, and recent technological advances have allowed imaging at a depth of >1 mm through the entire depth of the mouse cortex (Kobat et al., 2011; Horton et al., 2013). Spatial resolution of MPM *in vivo* imaging is typically 1 μm or better, which enables accurate imaging of all microvessels, including capillaries. However, the method is relatively slow, typically needing tens of minutes to acquire a three-dimensional angiogram over a ~1 mm field of view. Shadows beneath the large cortical surface vessels can cause discontinuities in the acquired data, and optical wave front distortions can impair imaging at larger depths. An example of a mouse cortical microangiogram obtained *in vivo* by MPM is shown in Figure 2 (Dorr et al., 2012). This study examined changes in cortical microvascular structure and function in a transgenic mouse model of Alzheimer's disease. Structural microvascular impairment (increased tortuosity and decreased diameter of the penetrating arterioles) was associated with accumulation of the amyloid-β and rescued by the administration of scyllo-inositol.

**Figure 3:**
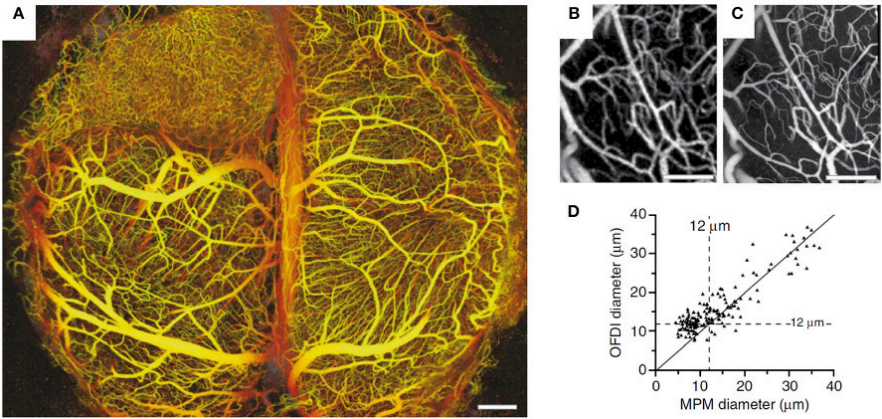
**(A)** The depth-projected vasculature within the first 2 mm of mouse brain bearing a xenotransplanted U87 human glioblastoma multiforme tumor imaged with OFDI. Depth is denoted by color: yellow (superficial) to red (deep). Scale bar, 500 μm. Right panels: Validation of the morphological measurements obtained from OFDI and MPM. **(B,C)** Normal brain vasculature acquired by OFDI **(B)** and MPM **(C)**. The automated vascular tracing was applied to registered vascular data sets to quantify the resolution of OFDI angiography and **(D)** validation of the morphological measurements obtained from OFDI. Scale bars, 250 μm. Adapted with permission from Vakoc et al. (2009).

In comparison with MPM, other modalities for high-resolution *in vivo* imaging of cortical microvasculature have been explored less. Among the other modalities, OCT has significant potential (Wang et al., 2007; Vakoc et al., 2009; Srinivasan et al., 2010). An optical analog of ultrasonic pulse-echo imaging (Drexler and Fujimoto, 2008), OCT detects scattering within the tissue to form cross-sectional images, and does not require exogenous contrast agents. Spatial resolution generally ranges from ~1 μm to a few tens of μm. Signals reflected from multiple locations along the optical axis (A-scans) can be acquired simultaneously, which gives OCT a significant speed advantage over MPM in volumetric imaging, with OCT microangiography taking several seconds compared with several minutes for MPM in comparable volumes of tissue. The typical penetration depth in rodent brain is ~1 mm (Srinivasan et al., 2011). The high-speed volumetric imaging is achieved by utilizing low numerical aperture (NA) imaging objectives, providing long depths of focus but reduced lateral spatial resolution. While OCT is currently subject to this tradeoff between lateral resolution and imaging depth, advances in hardware and processing algorithms may alleviate this limitation in the future (Leitgeb et al., 2006; Grulkowski et al., 2014; Mo et al., 2015). Shadows beneath large cortical surface vessels and optical wave front distortions at larger imaging depths are also present in OCT angiograms. In a study conducted by Vakoc et al. (2009), OCT microangiography was utilized to assess angiogenesis induced by a brain tumor. OCT provided microangiograms comparable to those obtained by MPM, including microvascular diameters (Figure 3).

PAI's contrast mechanism comes from the absorption of the excitation light by endogenous or exogenous chromophores, thereby creating heat. Images are formed by detecting ultrasonic waves generated by thermoelastic tissue expansion (Wang and Gao, 2014; Yao and Wang, 2014) The high-resolution application of PAI (optical-resolution photoacoustic microscopy, OR-PAM) relies on tight focusing of excitation light (Maslov et al., 2008) and can achieve a spatial resolution of several μm with ~1 mm penetration depth in a rodent brain (Hu et al., 2009; Wang et al., 2013a). The detection of ultrasonic waves in OR-PAM allows rapid acquisition of A-scans and, analogous to OCT, generation of three-dimensional images from two-dimensional scans.

Relative to *in vivo* microangiography, *ex vivo* imaging uses more invasive sample preparation and imaging approaches, and permits imaging of larger microvascular networks at high resolution (Yuan et al., 2015). Such methods include physical sample sectioning combined with confocal microscopy (Cassot et al., 2006; Lee et al., 2009) or two-photon microscopy (Blinder et al., 2013), tissue optical clearing (Chung and Deisseroth, 2013), and vascular casting with subsequent chemical tissue clearing and imaging with scanning electron microscopy (Konerding et al., 2001) or ionizing radiation (Plouraboue et al., 2004). Disadvantages of *ex vivo* microangiography include possible changes in vascular diameters compared to the *in vivo* state, difficulty in achieving complete filing of the vasculature when labeling the intravascular lumens, and spatial distortions due to sample preparation and/or physical sectioning.

### Segmentation of cerebral microvascular angiograms

Regardless of the imaging method used, the resulting data set typically consists of a volumetric intensity map. To provide a basis for analyzing functional properties such as blood flow or oxygen transport, this map must be converted into a defined vascular structure (Plouraboue et al., 2004; Wang et al., 2007; Reichold et al., 2009; Blinder et al., 2013; Yuan et al., 2015). Typically, this requires two main steps. First, the intravascular space must be distinguished from the extravascular space by segmentation, usually achieved setting an intensity threshold. From the results of this step, basic parameters such as vascular volume and vascular surface area can be deduced. Second, the intravascular space must be represented as a set of connected vessel segments, with identified lengths, diameters and nodal connection points. This information is required for the analysis of blood flow in the network, and provides a basis for modeling oxygen transport to tissue. Numerous vascular segmentation algorithms have been developed in recent years (Fridman et al., 2004; Kirbas and Quek, 2004; McIntosh and Hamarneh, 2006; Tyrrell et al., 2007; Shikata et al., 2009; Tsai et al., 2009; Rennie et al., 2011; Fraz et al., 2012; Hong et al., 2014). Detailed discussion of these algorithms is beyond the scope of this review.

Although the process of segmentation of the volumetric microvascular intensity images is simple in principle, it is subject to a number of practical difficulties. The spatial resolution of the imaging system is an important factor. If vessel boundaries are not sharply resolved, estimates of vessel diameters may be subject to substantial uncertainty. Furthermore, the choice of intensity threshold affects the region identified as being intravascular. According to Poiseuille's law, the flow rate in a vessel segment resulting from a given pressure drop varies as the fourth power of diameter. Predicted blood flow rates in microvascular networks are therefore very sensitive to estimated diameters. Variations in image contrast or intensity across the region of interest can lead to systematic deviations. In regions of low image intensity, some segments may fall below the chosen threshold, leading to incomplete mapping of the network and the appearance of multiple blind-ended vessels. The type of fluorescent labeling used for red blood cells, blood plasma or endothelial cells must also be considered when estimating vessel diameters. If a macromolecular plasma marker is used, the apparent diameter may be less than the anatomical diameter because large molecules may be excluded from the endothelial surface layer or glycocalyx, a layer up to about 1 μm thick of glycoproteins, polysaccharides and proteins that covers the inner surface of the vasculature (Pries et al., 2000; Vink and Duling, 2000).

The process of describing the vascular network in terms of segments and nodes, based on a three-dimensional map of the intravascular space, presents further challenges, particularly if the map is not of high quality. Available segmentation algorithms are unable to achieve high accuracy in an automated mode, and manual intervention by the user is often needed to achieve satisfactory results. This is highly time-consuming for cortical volumes such as those obtained *in vivo* by MPM (~700 × 700 × 700 μm^3^). Development of improved methods for analysis of three-dimensional *in vivo* microvascular images is a pressing need (Wang et al., 2013a; Leahy et al., 2015).

### Imaging of cerebral microvessel blood flows

Information on microvessel blood flow rates is essential for the analysis of oxygen delivery to cerebral tissue. Measurement of flow in all segments of extensive microvascular networks is currently not feasible. However, flow measurements in a subset of segments can be combined with theoretical models of blood flow in microvascular networks, permitting estimation of flow in all segments, as discussed below.

**Figure 4:**
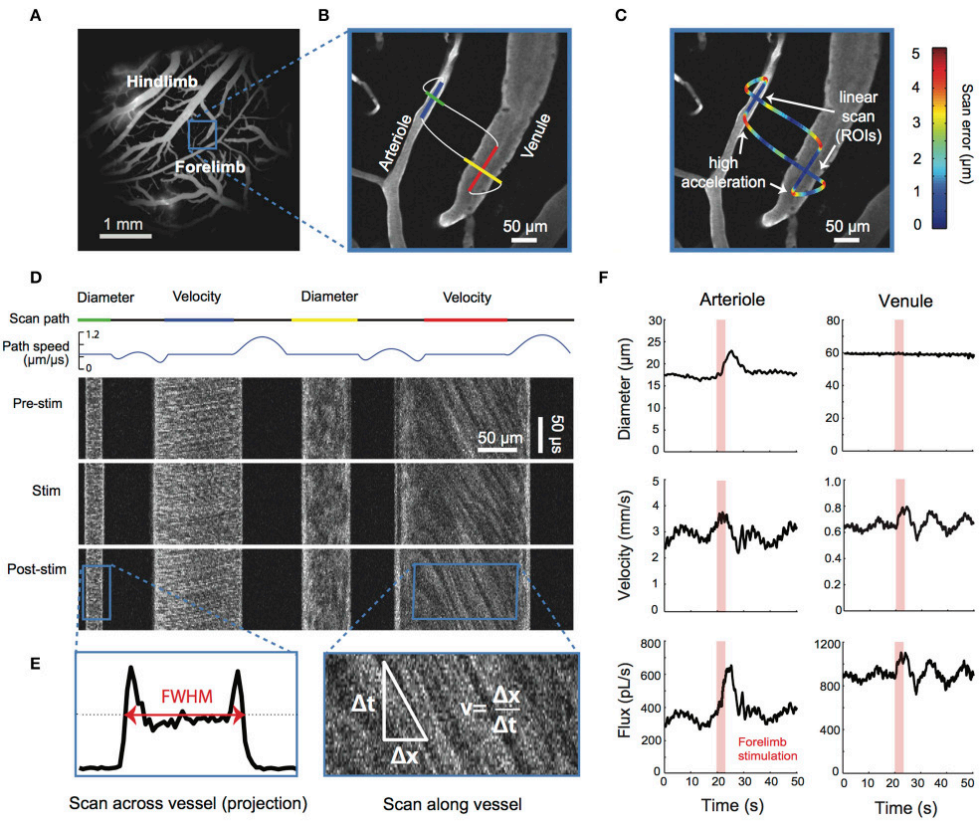
**TPM flow**. Simultaneous measurement of lumen diameter and red blood cell velocity in multiple vessels using spatially optimized line scans. **(A)** Image of fluorescein-dextran-labeled vessels in the rat somatosensory cortex taken with a 4X objective. **(B)** High-magnification image of a surface arteriole and venule in the forelimb region collected with a 40X objective. The pattern of the two-photon scanning laser is superimposed. Portions of the scan path along the length of the vessel are used to calculate red blood cell (RBC) velocity, whereas portions moving across the width of the vessels are used to calculate lumen diameter (Driscoll et al., 2011). Scans were acquired at a rate of 733 scan cycles per second. **(C)** The scan path is colored to show the error between the desired scan path and the actual path the mirrors traversed. The error along linear portions of the image is typically < 1 μm and increases when the mirrors undergo rapid acceleration. The error between successive scans of the same path is < 0.15 μm, several times lower than the point-spread function of a two-photon laser scanning microscopy. **(D)** The upper traces show the scan path and mirror speed as a function of time. Note that portions used to acquire diameter and velocity data are constant speed. The line scans generated from the path can be stacked sequentially as a space–time plot as shown in the lower image panels. Each image panel shows ~100 ms of data collected before, during, and after an electrical stimulation of the contralateral forelimb of the anesthetized rat. The stimulus was a 1 mA current, delivered for 3 s at 3 Hz with a 100-ms pulse width (Devor et al., 2007). **(E)** Vessel diameter is calculated as the full-width at half-maximum of a time-average of several scans across the width of a vessel (left). Red blood cell velocity is calculated from the angle of the RBC streaks (right; Drew et al., 2010). **(F)** Data traces of lumen diameter, RBC velocity, and RBC flux for the arteriole and venule after processing to remove heartbeat and smoothing with a running window. Figure adapted from Driscoll et al. (2011, Book chapter).

MPM can be used for high-resolution depth-resolved measurements of vessel diameters, RBC fluxes and flow velocities in small animal models (Kleinfeld et al., 1998; Kamoun et al., 2010; Shih et al., 2012; Figure 4). MPM utilizes fluorescent labeling of either plasma or RBCs and tracks RBC speed and flux in individual microvessels, including capillaries. There is a tradeoff between the number of simultaneously monitored vessels and temporal resolution. Fast blood flow transients that are characteristic of functional hyperemia or spreading depressions and require ~1 s imaging temporal resolution can be continuously monitored in only a few vessel segments at a time, while all vessels in the field of view can be sampled with ~12 s temporal resolution (Kamoun et al., 2010). However, multiplexing strategies for MPM are being explored (Ducros et al., 2013; Lecoq et al., 2014; Yang et al., 2016).

**Figure 5:**
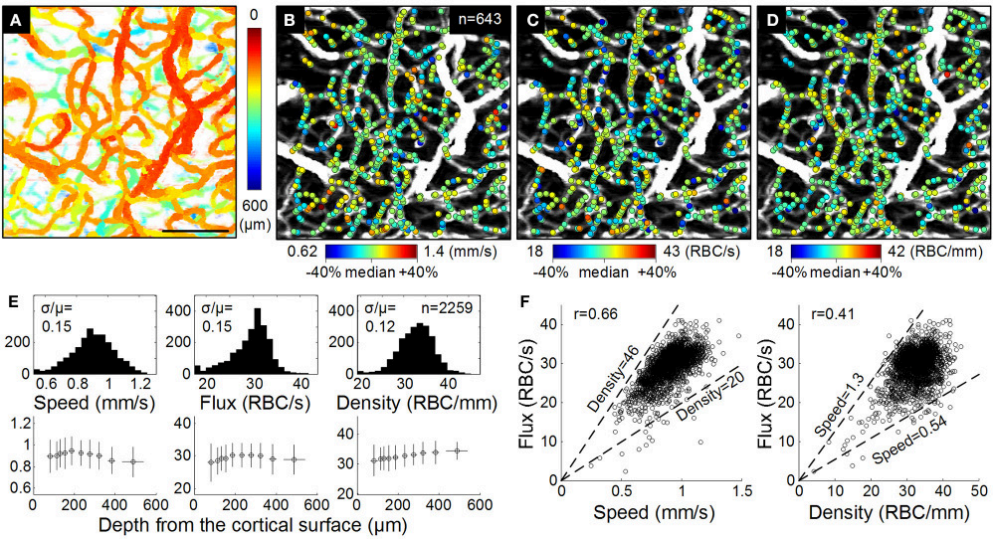
**OCT RBC flow. (A)** The enface MIP of the 3D angiogram with color indicating the depth from the cortical surface. Bar = 100 μm. **(B–D)** Estimated RBC speed, flux, and density are presented as color spots on the MIP angiogram. We used relatively comparable ranges (median ± 40%) for all quantities. **(E)** Histograms (top) and depth profiles (bottom) of the RBC speed, flux, and density measured from three animals (*n* = 2; 259 measures in total). Comparable histogram ranges (median ± 40%) are used. In the depth profiles, data are presented as mean ± s.d. **(F)** Correlations between the flux vs. the speed and density (*n* = 2259). Dashed lines indicate median ± 40% of the density (left) and speed (right). Adapted with permission from Lee et al. (2013).

OCT enables depth-resolved imaging of the absolute blood flow in individual cortical arterioles and venules (Wang et al., 2007; Wang and An, 2009; Srinivasan et al., 2010; Bouwens et al., 2013; Lee et al., 2013, 2014) as well as measurements of RBC flux in capillaries (Ren et al., 2012a; Srinivasan et al., 2012; Lee et al., 2013; Tokayer et al., 2013; Weiss et al., 2013; Figure 5). Compared to MPM measurements of blood flow, the advantages of OCT include increased penetration depth through the thinned skull in mice and >1 mm penetration depth through a cranial window, reliance on endogenous contrast (i.e., optical scattering) instead of exogenous contrast agents, and improved acquisition speed. OCT measurements of cortical blood flow have been validated (Srinivasan et al., 2011) and full volumetric imaging of blood flow over a cortical surface area of 1 mm^2^ is possible in ~1 min (Srinivasan et al., 2010). In addition to CBF measurements in non-capillary vessels, significant progress was made in the quantitative OCT measurement of capillary blood flow (Srinivasan et al., 2012; Lee et al., 2013, 2014). Volumetric images of capillary RBC flux can be obtained in a few minutes, opening the possibility of studying capillary perfusion in the brain and providing extensive empirical data for theoretical models of RBC flux distribution within capillaries. In combination with MPM, this technology will enable studies of the role of pericytes in CBF control. Commercial systems are available, facilitating widespread adoption of OCT. However, the data analysis for the OCT blood flow measurements is significantly more complex than for the MPM flow measurements. Therefore, widespread adoption of OCT flow measurements may also require the availability of sophisticated data processing tools. In short, while OCT has only recently emerged as a tool for studying brain CBF (Jia et al., 2009; Srinivasan et al., 2011; Ren et al., 2012b; Shen et al., 2014), its importance is expected to grow rapidly.

PAI can be also applied for measuring blood flow. By utilizing this technology, the characteristics of cerebral blood flow (CBF) can be measured using several different approaches, including blood velocity measurement in arterioles and venules based on Doppler broadening of the ultrasonic bandwidth (Yao et al., 2010), combining ultrasonic thermal tagging with PAI (Wang et al., 2013b), and even single RBC velocity measurement (Wang et al., 2013a). A combination of PAI measurements of cortical blood flow and oxygen saturation may allow estimation of local oxygen consumption rates.

### Imaging of cerebral oxygenation

Theoretical models can be used to predict the distribution of tissue oxygen levels surrounding a microvascular network, as discussed later. However, direct measurement of tissue and/or blood oxygen levels is needed to provide boundary conditions for such models, to allow estimation of unknown parameters, and to test the resulting predictions. A large body of experimental studies have established the basic properties of intra- and extravascular oxygen changes during increases in neuronal activity and associated functional hyperemia (vasodilation and an increase in CBF and volume) (Vanzetta and Grinvald, 1999; Vovenko, 1999; Ances et al., 2001; Erecińska and Silver, 2001; Masamoto et al., 2003; Thompson et al., 2003; Offenhauser et al., 2005; Viswanathan and Freeman, 2007; Sharan et al., 2008; Yaseen et al., 2009; Vazquez et al., 2010). However, only recently several optical microscopy imaging technologies were developed opening the door for microscopic *in vivo* imaging of intravascular and tissue oxygenation with unprecedented spatial and temporal resolution within cortex.

**Figure 6:**
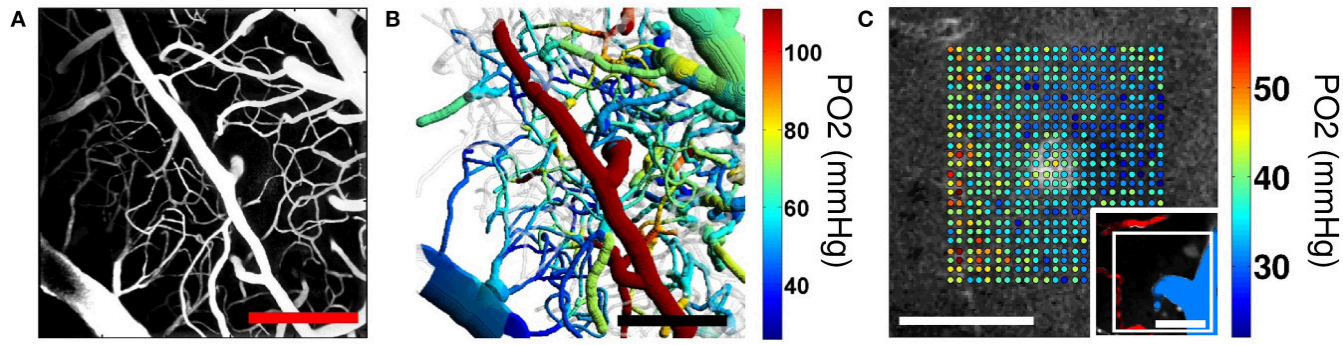
**Two-photon microscopy imaging of PO_**2**_. (A)** Maximum intensity projection (MIP) of the 200−μm-thick cortical microvascular stack obtained by TPM. Blood plasma was labeled by FITC. **(B)** Top-view projection of the segmented microvasculature with the intravascular PO_2_ measurements obtained by TPM. Mean vascular segment PO_2_ measurements were color-coded and overlaid on the segmented microvascular structure. Scale bars, 200 μm. Adapted with permission from (Sakadžić et al., 2014). **(C)** Simultaneous measurement of PO_2_ in cortical vasculature and tissue. Measured PO_2_ values overlaid with the gray scale phosphorescence intensity image at 60 μm depth. Measurements were performed at the location of an ascending venule. Measurement location is marked with the white rectangle in the inset (bottom right), showing MIP of 80 μm-thick FITC-labeled microvasculature stack. Ascending venule (blue) and branches of the descending arteriole (red) are color-coded for easier identification. Scale bars, 50 μm. Adapted with permission from Sakadžić et al. (2010).

Two-photon microscopy-based phosphorescence lifetime imaging (PLIO_2_) allows mapping intravascular and tissue partial pressure of O_2_ (PO_2_) with micrometer spatial resolution (Finikova et al., 2008; Sakadžić et al., 2010; Lecoq et al., 2011; Figure 6). The technique measures oxygen-dependent phosphorescence lifetimes of an exogenous contrast agent (Vanderkooi et al., 1987; Rumsey et al., 1988). The phosphorescence lifetime of a probe depends on the PO_2_ in the immediate vicinity of the probe, providing a spatially localized measurement of dissolved oxygen. Probe molecules for MPM were specially designed for two-photon excitation, with a high degree of encapsulation that ensures stability of the lifetime calibration in a complex biological environment (Finikova et al., 2008; Lebedev et al., 2009; Roussakis et al., 2014). Unlike spectroscopy-based hemoglobin saturation measurements, PLIO_2_ lifetime imaging is insensitive to changes in tissue optical properties during imaging. The acquisition speed is currently limited to 0.2–0.5 s per measurement point by relatively long phosphorescence lifetimes and the number of decay averages required at each point. Further development of oxygen sensitive dyes with significantly higher quantum yield, two-photon absorption cross section, and dynamic range will enable significant improvements in acquisition speed and precision of PO_2_ imaging (Esipova and Vinogradov, 2014). This technology has been used to obtain high-resolution maps of oxygen concentration distribution in both the microvasculature and tissue under various conditions (Devor et al., 2011; Kazmi et al., 2013; Parpaleix et al., 2013; Sakadžić et al., 2014; Spencer et al., 2014) and the awake mouse brain (Lyons et al., 2016).

**Figure 7:**
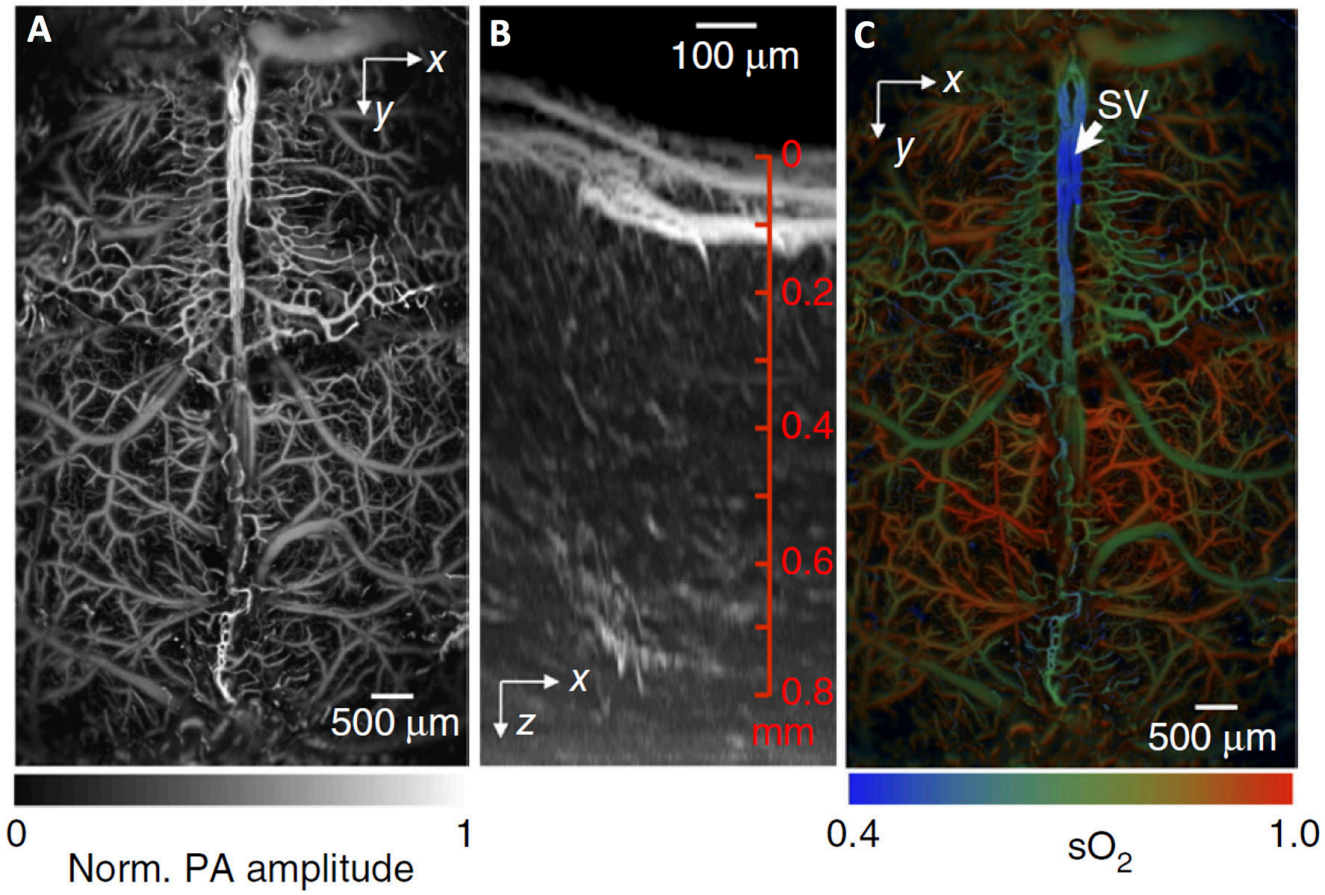
**Fast functional photoacoustic microscopy (PAM) of the mouse brain. (A)** Representative xy projected brain vasculature image through an intact skull. **(B)** Representative enhanced xz projected brain vasculature image acquired over a 0.6 × 0.6 mm^2^ region with depth scanning, where the signal amplitude was normalized depthwise. **(C)** PAM of oxygen saturation of hemoglobin (SO_2_) in the same mouse brain as in **(A)**, acquired by using the single-wavelength pulse-width-based method (PW-SO_2_) with two lasers. SV, skull vessel. Adapted with permission from Yao et al. (2015).

Intravascular oxygenation can be also measured with PAI because hemoglobin is the dominant optical absorber of visible and near-infrared radiation in the brain. PAI provides quantification of both oxygen saturation and total hemoglobin concentration (Figure 7). In particular, high spatial resolution variants of PAI can achieve ~1 mm penetration depth in the brain and were successfully applied to measure arteriolar, venular and capillary oxygen saturation (SO_2_) as well as single RBC oxygenation (Wang et al., 2013a; Yao and Wang, 2014). Recently, high acquisition rate PAI was used to image the hemodynamic response to hind limb stimulation in the mouse somatosensory cortex (Yao et al., 2015). PAI has also been applied to image brain pathologies including edema after cold injury (Xu et al., 2011), and dynamics of the microvascular oxygenation after middle cerebral artery occlusion (Hu et al., 2011) and during epileptic seizure (Tsytsarev et al., 2013). The ability of PAI to measure RBC oxygen saturation and flux in capillaries has potential applications for analyzing capillary flow and oxygenation under various conditions.

**Figure 8:**
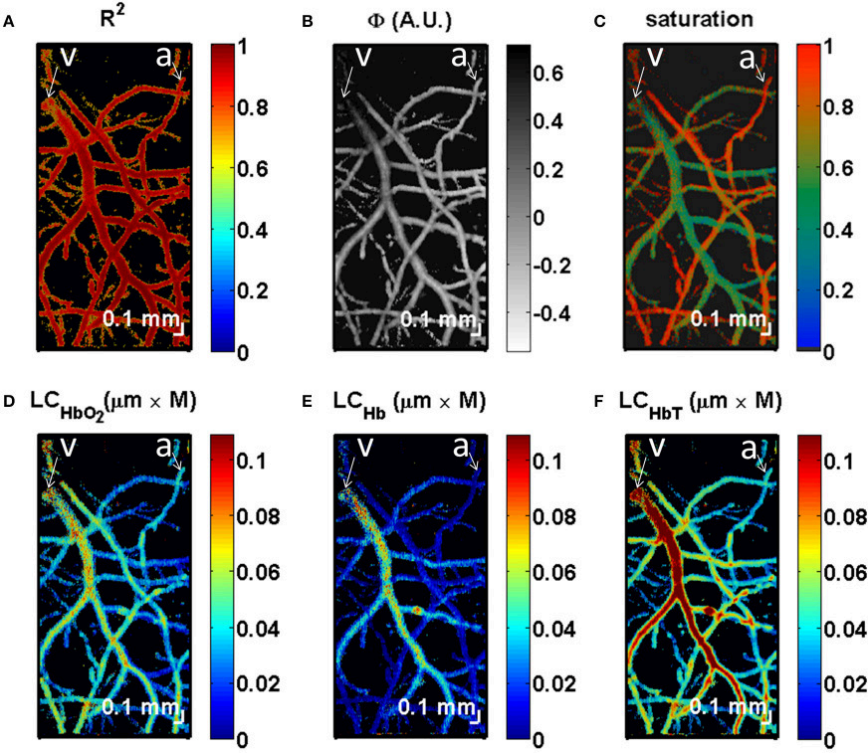
**OCT measurement of SO_**2**_**. Quantification of chromophores in the mouse brain in an *en face* view. **(A)** Maximum intensity projection of *R2* values from the fit (Equation 19) shows the highest values near the centers of vessels, with a decrease at the edges. **(B)** The parameter Φ accounts for RBC scattering effects. **(C)** Saturation map, showing clear distinctions between arteries and veins. **(D)** Map of the maximum of the product of oxygenated hemoglobin concentration and distance shows that veins and arteries contain oxyhemoglobin. **(E)** By comparison, under the given experimental conditions, most of deoxyhemoglobin is contained in the veins. **(F)** The map of the maximum of the product of total hemoglobin concentration and distance shows larger values in larger vessels, with localized increases at vessel crossings. It should be noted that quantitative measurements of chromophores can be achieved by integrating the maps **(D–F)** in the transverse plane (*x* and *y* dimensions). All maps were displayed with transparency based on the local *R2* values at each transverse location, averaged over depth. An artery (a) and vein (v) are labeled. Adapted with permission from Chong et al. (2015b).

Another imaging technique, visible light OCT (vis-OCT), is emerging as an alternative that may provide rapid non-invasive measurements of microvascular oxygenation (Robles et al., 2011; Pan et al., 2014; Chong et al., 2015a). This will permit measurements of tissue and vascular structure, blood flow and oxygen transport, all obtained using OCT (Figure 8).

## Theoretical modeling of cerebral microvascular hemodynamics and oxygen transport

### Blood flow simulation

Analysis of oxygen transport in a microvascular network requires knowledge of blood flow and red blood cell (RBC) fluxes in each vessel segment, since they govern the convective transport of oxygen. To model microvascular hemodynamics, the network is generally represented as a set of interconnected cylindrical segments, in which each segment is characterized by its conductance, defined as the ratio of volume flow rate (*Q*) to pressure drop (ΔP) (Lipowsky and Zweifach, 1974). According to Poiseuille's law, the conductance is given by π*d*^4^/(128*L*μ), where *d* is the diameter, *L* is the length and μ is the viscosity of blood. This formula shows that conductance is strongly dependent on vessel diameter: a small increase in diameter leads to a significant flow increase. In network flow simulations, small inaccuracies in diameters derived from image processing can therefore significantly affect the predicted flow distributions. The effective viscosity of blood, μ, has been determined experimentally, and varies strongly with vessel diameter as a result of the particulate nature of blood (the Fåhraeus-Lindqvist effect). Empirical equations derived from experimental observations give apparent blood viscosity as a function of vessel diameter and hematocrit (Pries et al., 1992, 1994). These equations take into account the inhomogeneous characteristics of blood flow in microvessels, including the formation of a cell-free or cell-depleted layer near vessel walls, resulting from the fact that it is a concentrated suspension of RBCs. The effects of the endothelial surface layer adjacent to the vessel walls are also included.

As a result of the particulate nature of blood, the partition of RBC flux at diverging microvessel bifurcations generally differs from the partition of plasma flow. This implies that the hematocrit in each daughter vessel may differ from that in the parent vessel. Empirical equations have been developed to describe this “phase separation” effect, including its dependence on the flow split in the bifurcation, the vessel diameters, and the discharge hematocrit in the parent vessel (Pries et al., 1989; Pries and Secomb, 2005). The discharge hematocrit (*H*_*D*_) is defined as the volume flux of RBCs as a fraction of the total volume flow rate, and should be distinguished from the tube hematocrit (*H*_*T*_), which is defined as the fraction of the vessel volume occupied by RBCs. Generally *H*_*D*_ is larger than *H*_*T*_, because RBCs preferentially flow near the vessel center-line where the velocity is higher, a phenomenon known as the Fåhraeus effect. According to the above definitions, the volume flux of RBCs is given by *QH*_*D*_, where *Q* is the volume flow rate of the segment. The empirical equations referred to above for phase separation refer to situations where a single vessel splits into exactly two branches. An alternative approach, which has the advantage of applicability to branch points with more than two outflowing segments, has been proposed (Gould and Linninger, 2015b). This approach excludes the possibility of zero hematocrit in low-flow branches, whereas such behavior can be observed *in vivo*, and fits experimental data less closely than the earlier empirical equations (Pries et al., 1989; Gould and Linninger, 2015a). A further alternative approach has been proposed based on the assumption that the distribution of RBCs in diverging bifurcations satisfies an optimality principle (Sriram et al., 2014).

**Figure 9:**
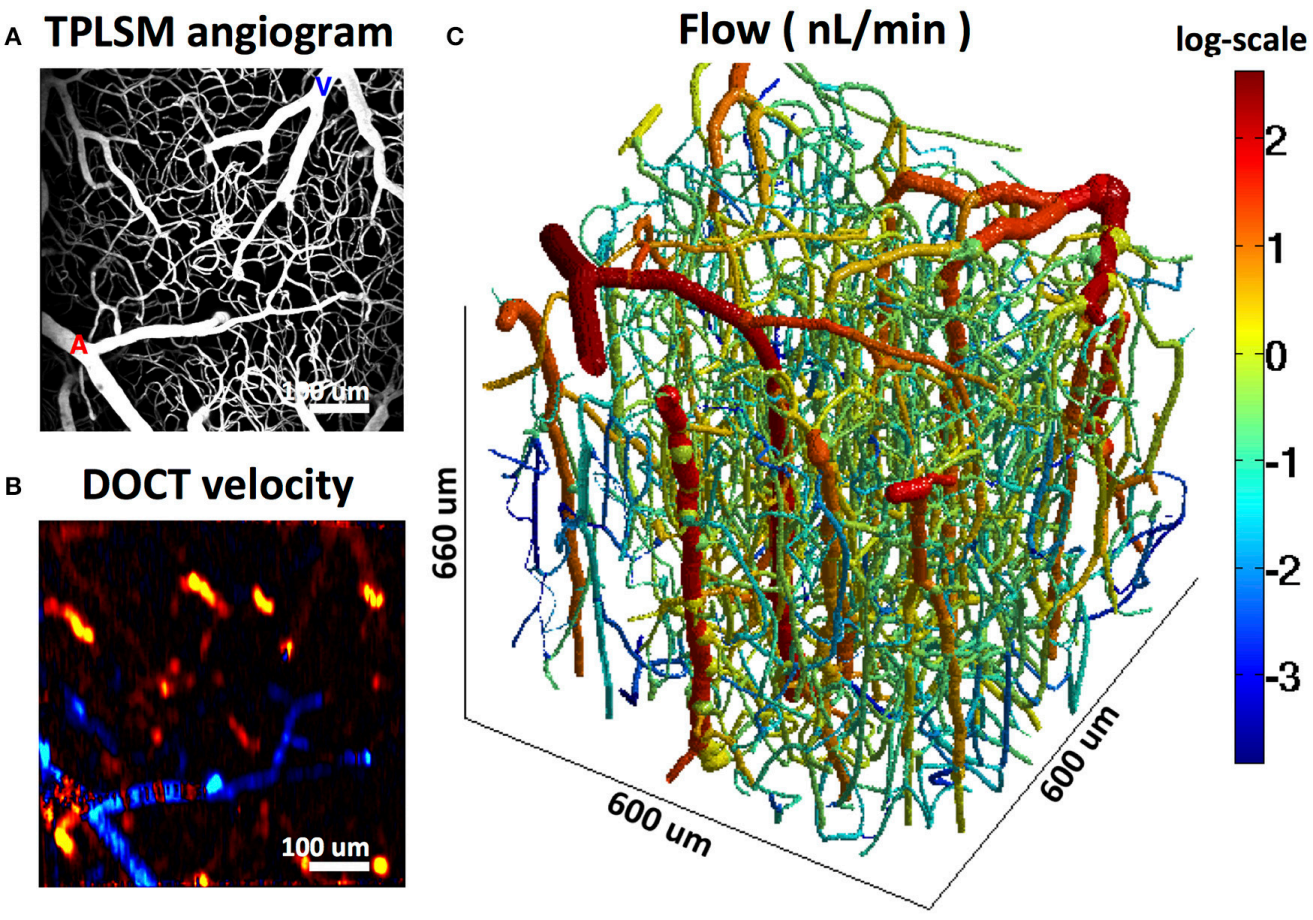
**Reconstruction of microvascular cerebral blood flow in the mouse cortex using Doppler OCT flow measurements in outflowing venules to constrain the computation. (A)** MPM angiogram. **(B)** Doppler OCT velocity projection map. **(C)** Reconstructed flow. Adapted with permission from Gagnon et al. (2015a).

When network models of this type are applied to observed microvascular network structures, the problem arises that many vessels typically cross the boundaries of the sampled region. To obtain a unique flow solution, it is necessary to impose flow or pressure boundary conditions (BCs) on these inflowing and outflowing segments, and hematocrit boundary conditions on inflowing segments. Not all these flow or pressure BCs are generally available from experimental observations, although a subset may be available. Several approaches have been used to address this difficulty. Lorthois et al. (2011a,b) considered two regimes: (i) uniform pressure BCs applied to boundary capillaries, with a pressure value chosen to give zero net flux into the capillaries; (ii) zero flow conditions imposed at boundary capillaries. These two cases gave upper and lower bounds on the total network perfusion. The latter approach was also used by Gagnon et al. (2015a,b). Fry et al. (2012) developed an approach to address this problem by solving for the flows throughout the network while minimizing the deviation from an expected “target” pressure and shear stress in each vessel segment. These target values must be estimated based on observations of typical values in the tissue under consideration. In an analysis of flow distribution in cortical networks (Gagnon et al., 2015a), pressure boundary conditions were imposed using literature values based on vessel diameters. The computation was constrained using measurements of flow in a subset of vessels (Figure 9). This was shown to reduce the impact of pressure boundary conditions and estimated vessel resistances. The error in flow computation was reduced by 50% when flow measurements in major outflowing vessels were included in the set of constraints.

### Models for oxygen transport

Oxygen transport takes place over a range of spatial scales and involves multiple biophysical processes. The challenge of developing theoretical models for oxygen transport to tissue has stimulated a variety of approaches. Here we briefly review the development of the field, focusing on cerebral microcirculation. For a more detailed review, see Goldman (2008).

A key early contribution was a model inspired by the somewhat regular structure of skeletal muscle (Krogh, 1919). The Krogh cylinder model assumes an array of parallel, evenly spaced capillaries, each of which supplies oxygen to a tissue cylinder surrounding it, with a fixed rate of consumption. This leads to a simple steady-state reaction-diffusion equation, through which the radial variation in tissue PO_2_ can be computed as a function of distance from the vessel. The Krogh model has been extended to include other effects including time-dependent transport, PO_2_-dependent oxygen consumption according to Michaelis-Menten kinetics, myoglobin-facilitated tissue transport and intravascular resistance to radial oxygen diffusion. Hudetz et al. (1999) adapted the Krogh model to study oxygenation in the cerebral cortex, assuming the capillary supplied a conical tissue region that tapered downstream, such that venous capillaries supply smaller regions. This model exhibited a non-linear relation between consumption and blood flow, i.e., for a significant rise in consumption rate, the blood flow increase required to avoid hypoxia was disproportionately larger.

Using a finite-element model of oxygen transport by discrete moving RBCs, Lücker et al. (2015) analyzed the transient variation in tissue oxygenation due to the passage of RBCs and the difference in PO_2_ between RBCs and plasma. These variations have been termed erythrocyte-associated transients (EAT). The authors suggested that a higher capillary density on the venular side of the network must be present to ensure tissue oxygenation. Network-scale models of oxygenation including transient effects associated with each RBC would be very expensive computationally. An approximate approach based on an effective blood PO_2_ is generally used, as described in the next section.

In general, Krogh-type approaches are appropriate for muscle, particularly under conditions of high oxygen demand, but do not provide a good approximation in situations where the geometry is not approximated by parallel capillaries. The Krogh model overestimates minimum tissue PO_2_ and thus fails to predict the onset of hypoxia in such geometries (Secomb et al., 1993, 2000). Therefore, approaches have been developed for modeling oxygen delivery by networks of vessels. One relatively simple approximate approach is to treat the extravascular space as a well-mixed compartment with respect to oxygen transport, with the resistance to oxygen transport residing in the vessel wall (Jespersen and Østergaard, 2012; Angleys et al., 2015). Finite element or finite difference methods have been employed to simulate oxygenation in arrays of parallel capillaries (Hoofd, 1995; Goldman and Popel, 2000; Beard and Bassingthwaighte, 2001), networks with interconnected parallel capillaries (Wieringa et al., 1993; Goldman and Popel, 2000; Beard and Bassingthwaighte, 2001), synthetic networks generated based on measured distributions of anatomical properties (Lauwers et al., 2008; Linninger et al., 2013; Safaeian and David, 2013; Park and Payne, 2016) and networks extracted from three-dimensional angiograms (Fang et al., 2008; Gagnon et al., 2015b). An efficient computational method applicable to arbitrary vascular network geometries has been developed using a Green's function approach (Hsu and Secomb, 1989; Secomb et al., 2004), as discussed later.

### Intravascular oxygen transport

The rate of oxygen transport along a microvessel by convection is given by:

(1) $$\begin{array}{l} J\left(P_{b}\right)=Q\left[H_{D}C_{0}S\left(P_{b}\right)+H_{D}\alpha_{rbc}P_{b}+\left(1-H_{D}\right)\alpha_{pl}P_{b}\right] \end{array}$$

where *P*_*b*_ is the PO_2_ in the blood, *S* is the fractional oxyhemoglobin saturation, *H*_*D*_ is the discharge hematocrit, *C*_0_ is the concentration of hemoglobin-bound oxygen in a fully saturated RBC and α_*pl*_ and α_*rbc*_ are the solubilities of oxygen in plasma and RBCs respectively. This may be simplified to give:

(2) $$\begin{array}{l} J\left(P_{b}\right)=Q\left[H_{\text{D}}C_{0}S\left(P_{b}\right)+\alpha_{\text{eff}}P_{b}\right] \end{array}$$

where α_eff_, the effective solubility of oxygen in blood, depends on the discharge hematocrit *H*_*D*_ and on the solubilities in plasma and red blood cells. Under normal conditions, the majority of transported oxygen is bound to hemoglobin in the RBCs. The contribution of dissolved oxygen to convective oxygen transport is normally small, but may become significant if *P*_*b*_ is very high or *H*_*D*_ is very low. The binding of oxygen to hemoglobin is a complex process which depends on several factors including blood pH, temperature and CO_2_ concentration. However, the saturation can be fairly accurately represented by the Hill equation:

(3) $$\begin{array}{l} S\left(P_{b}\right)=P_{b}^{n}/\left(P_{50}^{n}+P_{b}^{n}\right) \end{array}$$

where *P*_50_ is the PO_2_ at 50% saturation, which is in the range 29–40.5 mmHg depending on the species, and *n* is the Hill exponent, which is typically in the range 2–3 (Ellsworth et al., 1988; Uchida et al., 1998; Ellis et al., 2002). The value of *P*_50_ is important in determining the critical range of PO_2_ values for oxygen unbinding, due to the sharp gradient in the Hill equation curve around this value. Conservation of mass implies that:

(4) $$\begin{array}{l} dJ\left(P_{b}\right)/ds=-q_{\text{v}}\left(s\right) \end{array}$$

where *s* is distance along the segment and *q*_*v*_ is the rate of diffusive oxygen efflux per unit vessel length. In simulations of oxygen delivery by microvascular networks, the above equations are applied to each segment in the network.

The above equation for convective oxygen flux *J* assumes that the blood PO_2_ at each point along the vessel is described by a single value, *P*_*b*_. In reality, significant gradients in PO_2_ occur within the vessel cross-section, associated with the radial diffusion of oxygen from blood to tissue (Hellums, 1977). Therefore, the quantity *P*_*b*_ represents an effective average blood PO_2_, such that the convective oxygen flux is correctly specified.

### Oxygen diffusion and consumption in tissue

If the diffusivity *D* and solubility α of oxygen in the tissue are uniform, the tissue PO_2_, *P*(*x, y, z*) satisfies:

(5) $$\begin{array}{l} D\alpha \nabla^{2}P=M\left(P\right) \end{array}$$

under steady-state conditions, where ∇^2^ is the Laplacian operator and *M*(*P*) is the oxygen consumption rate. Experimentally, oxygen consumption rates are approximately constant above a certain tissue PO_2_ value, but drop sharply below this. This behavior is commonly represented by Michaelis-Menten kinetics:

(6) $$\begin{array}{l} M\left(P\right)=M_{0}P/\left(P+P_{0}\right), \end{array}$$

where *M*_0_ is the rate when oxygen is not rate-limiting (maximal consumption rate), and *P*_0_ is the PO_2_ value at half-maximal oxygen consumption. In previous work, this has usually been assumed to be about 1 mmHg, but recent experimental results put it closer to 5–10 mmHg (Golub and Pittman, 2012).

At the blood-tissue interface, the tissue oxygen field *P*(*x, y, z*) must satisfy boundary conditions representing continuity of diffusive oxygen flux and continuity of partial pressure. Continuity of flux implies that:

$$\begin{array}{l} q_{v}\left(s\right)=-D\alpha a\overset{2\pi }{\underset{0}{\int }}\frac{\partial P}{\partial r}d\theta \end{array}$$

where *r* is radial distance from the vessel centerline, *a* is the vessel radius and the integral is around the vessel circumference. The matching of PO_2_ values at the interface requires consideration of the radial intracapillary gradients, mentioned earlier. Since these gradients drive the radial flux of oxygen, they can be assumed to be proportional to the rate of oxygen loss from the vessel. This relationship can be expressed as

(7) $$\begin{array}{l} P_{v}\left(s\right)=P_{b}\left(s\right)-Kq_{v}\left(s\right) \end{array}$$

where *P*_*v*_(*s*) is the tissue PO_2_ averaged around the circumference of the vessel and *K* represents intravascular resistance to radial oxygen transport. This resistance parameter shows a strong dependence on vessel diameter (Hellums et al., 1996) and depends on hematocrit (Roy and Secomb, 2014). As a result of these matching conditions, the equations for oxygen transport by convection in the vessels and by diffusion in the tissue are strongly coupled and must be solved simultaneously. This is a challenging problem when large networks containing many segments with a complex geometry are considered.

### Green's function methods

One approach to address this challenge is to represent the tissue oxygen field as a superposition of fields resulting from a distribution of oxygen sources (the vessel segments) and oxygen sinks (consumption in the tissue). In an infinite domain, under steady-state conditions, the PO_2_ field resulting from a unit point source of oxygen is given by:

(8) $$\begin{array}{l} P=1/\left(4\pi D\alpha r\right) \end{array}$$

where *r* is the distance from the source. This is the Green's function for the three-dimensional Laplacian operator. This approach permits computation of spatially resolved oxygen fields for complicated vascular network structures with a reasonable computational cost (Hsu and Secomb, 1989; Secomb et al., 2004) and has been applied to a various tissues including brain (Secomb et al., 2000). The Green's function approach uses an iterative solution method and can be applied when the reaction rate kinetics are non-linear, as in the case of Michaelis-Menten kinetics, or the solute binding characteristics in blood are non-linear, as in the case of the Hill equation for oxygen binding to hemoglobin. An analogous approach has also been developed for time-dependent diffusion problems (Secomb, 2015).

## Applications and future directions

### Estimation of microvascular blood flow

As already described, current imaging approaches can provide three-dimensional maps of extensive network structures, including vessel diameters, together with flow rate measurements in a subset of segments. Theoretical models can then be used to reconstruct the entire flow distribution. The limitation of incomplete flow information for boundary segments can be addressed by constraining flows in selected segments (Figure 9) to match measured values (Gagnon et al., 2015a) or by using an optimization approach based on typical distributions of pressures and shear rates (Fry et al., 2012). Combining these two methods represents a logical future development. The emergence of new imaging modalities for measuring flow in capillaries, such as OCT (Srinivasan et al., 2012; Lee et al., 2013, 2014), MPM (Kleinfeld et al., 1998; Desjardins et al., 2014) and PAM (Wang et al., 2013a) will provide flow data for more vessel segments, and therefore allow more accurate flow reconstructions. Improvements in acquisition speed will allow for dynamic measurements during brain activation, which will permit studies aiming to improve interpretation of fMRI signals that are routinely used in human neuroscience studies (Bouchard et al., 2015).

In a study of blood flow in the microvessel networks of the rat mesentery, Pries et al. (1994) showed that the apparent viscosity of blood differed strongly from that in glass tubes of corresponding diameters. The main cause of this discrepancy was found to be the presence of a relatively thick (~1 μm) glycocalyx or endothelial surface layer (Pries et al., 2000). However, it is not known to what extent this effect occurs in other tissues, including brain. The methods discussed here provide a potential approach for investigating the hemodynamic effects of the ESL in brain microcirculation.

### Assessment of tissue oxygenation

Overall oxygen transport rates to cortical tissue can be estimated from tissue-level parameters. For instance, the rate of oxygen consumption per unit volume is given by:

(9) $$\begin{array}{l} \text{CMR}{\text{O}}_{2}=\text{Ca}{\text{O}}_{2}\times \text{CBF}\times \text{OEF}, \end{array}$$

where CaO_2_ is the arterial oxygen concentration and OEF is the oxygen extraction fraction given by:

(10) $$\begin{array}{l} \text{OEF}=\left(\text{Sa}{\text{O}}_{2}-\text{Sv}{\text{O}}_{2}\right)/\text{Sa}{\text{O}}_{2}. \end{array}$$

and SaO_2_ and SvO_2_ are the oxygen saturations in arteries and veins, respectively. With the development of cortical measurements of PO_2_, SO_2_, and CaO_2_ with MPM (Sakadžić et al., 2010; Lecoq et al., 2011), PAM (Yao et al., 2013), and OCT (Chong et al., 2015b), SaO_2_ and SvO_2_ can be measured *in vivo* and OEF can be computed from these parameters, allowing assessment of local variations in these quantities.

**Figure 10:**
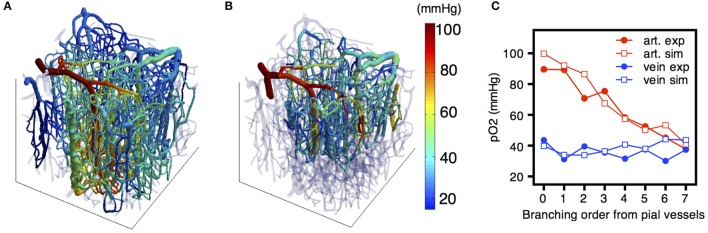
**(A)** Distribution of the partial pressure of oxygen (PO_2_) simulated across the vascular network using the FEM model. **(B)** MPM experimental measurement of PO_2_ *in vivo*. **(C)** Quantitative comparison of simulated and experimental PO_2_ and SO_2_ distributions across the vascular network. Adapted with permission from Gagnon et al. (2015b).

However, these parameters are not sufficient to allow estimation of tissue oxygen levels. Due to the heterogeneous structure of the microcirculation and the significant gradients in PO_2_, the spatial frequency distribution of tissue PO_2_ is much wider than would be the case in an ideal structure such as a Krogh cylinder (Secomb et al., 2000). Theoretical models for oxygen transport are needed to compute the spatially resolved tissue oxygen field (Secomb et al., 2000, 2004; Fang et al., 2008; Linninger et al., 2013; Gagnon et al., 2015b). However, the predictions of such models depend on oxygen consumption rate, oxygen levels in inflowing vessels, vessel flow rates, and other parameters that are not accurately known. A combination of modeling and measurements is needed to constrain these parameters (Gagnon et al., 2015b; Figure 10). The increasing availability of micro-scale measurements of tissue and vessel PO_2_, combined with the computationally efficient Green's function method for simulating oxygen transport, will make possible increasingly detailed and robust predictions of network-scale tissue PO_2_ distributions under a range of normal and pathological conditions.

### Modeling of neurovascular coupling and fMRI signals

On the macroscopic level of vascular dynamics, a number of theoretical models have been developed to describe the active regulation of blood flow to the brain. Among them, Ursino and Lodi (1998) used a compartmental model to analyze the regulation of CBF in response to changes in arterial pressure, intracranial pressure and carbon dioxide level. Other models such as the Balloon model (Buxton et al., 1998) or the double-compliance model (Mandeville et al., 1999) have also been developed.

The mechanisms of blood flow regulation and neurovascular coupling at the microscopic level are still not fully understood. Recently, a number of groups produced controversial evidence regarding the role of pericytes in controlling dilation and constriction of the capillary bed (Hall et al., 2014; Hill et al., 2015). The population of pericytes is heterogeneous, and not all of them are contractile as documented by many groups in various tissues including brain (Armulik et al., 2011; Attwell et al., 2015; Hartmann et al., 2015; Trost et al., 2016). There is a general agreement that high-branching-order capillaries cannot actively dilate or constrict (Hall et al., 2014; Hill et al., 2015). The debate is focused on low order branches off diving arterioles in a mouse cortex and whether these branches should be considered capillaries or precapillary arterioles. Hall at al. classified all branches off diving cerebral arterioles as capillaries based on the presence of pericytes. However, pericytes are present also on top of the smooth muscle wall in arterioles including larger pial arteries. Therefore, capillary definition based on the presence of pericytes may be ambiguous. Two-photon imaging indicates that the first couple of branching orders off diving cerebral arterioles are noticeably thicker that the average high order capillary supporting the existence of precapillary arterioles in between diving arterioles and the capillary bed. Regardless of nomenclature issues, there is a consensus across studies that these low-order branches (which may include true capillaries) can dilate and constrict (Tian et al., 2010; Hall et al., 2014; Uhlirova et al., 2016).

The distinction between precapillary arterioles and capillaries at sites of control is functionally relevant because terminal arterioles typically supply several capillaries, and so do not provide control at the individual capillary level. Also, an arteriole is spatially separated from the capillaries that it feeds, implying the need for mechanisms to coordinate arteriolar contraction with metabolic needs at the capillary level. The modeling approaches described above can be used to investigate the implications of different proposed mechanisms of neurovascular coupling. For example, network models can be used to investigate the effects of local vascular dilation on flow distribution (Reichold et al., 2009). Development of spatially resolved network-level models of neurovascular coupling is at an early stage and represents an important challenge for future work. Complexity arises from the fact that multiple biological mechanisms are involved. The roles of pressure-dependent (myogenic), shear-dependent and metabolic responses, and of conduction of signals along the vessel wall, have been explored in an integrated model for flow regulation in a simplified vascular network in skeletal muscle (Arciero et al., 2008; Carlson et al., 2008). In the brain, the roles that potassium ion fluxes in synaptic regions, astrocytes and arterioles play in vasodilation have been investigated theoretically (Witthoft et al., 2013). Progress in developing spatially resolved models for neurovascular coupling, network blood flow and oxygen transport has potential to lead to better understanding of the mechanisms determining CBF and oxygen transport (Boas et al., 2008; Fang et al., 2008) and to provide a basis for bottom-up modeling of fMRI signals (Gagnon et al., 2015b).

**Figure 11:**
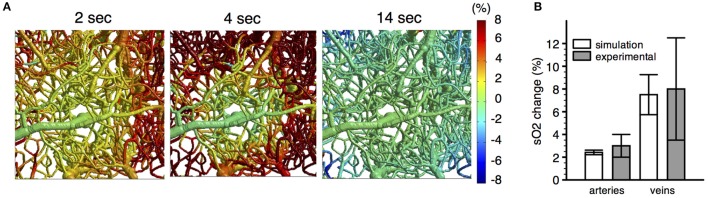
**(A)** Spatiotemporal evolution of SO_2_ changes following a 2 s forepaw stimulus. **(B)** Comparison of simulated SO_2_ changes (*n* = 6 animals) with experimental SO_2_ changes (*n* = 10 animals) measured in pial vessels during a forepaw stimulus with confocal microscopy. Adapted with permission from Gagnon et al. (2015b).

As a step in this direction, the experimental and theoretical methods reviewed here provide a basis for modeling the effects of prescribed changes in vascular diameter on blood flow and tissue oxygenation (Figure 11). For instance, Gagnon et al. (2015b) used experimental time courses of arterial dilation obtained with MPM (Tian et al., 2010) as a basis for simulating the resulting changes in blood flow and tissue oxygenation. Given the dynamics of CBF in the vasculature and the time course of CMRO_2_ in the cortical tissue, the dynamic distribution of SO_2_ was computed (Figure 11). Good agreement was obtained between the simulations and the experimental confocal microscopic measurements of SO_2_ during functional activation. The results of such simulations can be extended to model fMRI signals from first principles with Monte Carlo simulations of proton diffusion. This approach allowed quantification of the compartmental microvascular origin of BOLD-fMRI for different magnet strengths and MR pulse sequences with high accuracy. This approach also predicted that the BOLD response measured in human studies is influenced by the local folding of the cortex and its orientation in the magnet, a prediction that was confirmed by measuring the amplitude of the BOLD response to hypercapnia for different cortical orientations (Gagnon et al., 2015b).

## Conclusion

Progress in understanding neurovascular function (or its dysfunction in disease) requires experimental technologies and computational tools to accurately account for spatially distributed and dynamic processes of vasodilation/constriction and O_2_ transport. These tools—on their own—will not be sufficient for reconstruction of the underlying neuronal activity. Eventual progress toward this goal, however, will not take place without understanding the normal vascular network dynamics and how it is altered in disease. In the future, coupling these realistic 3D vascular network models to large-scale models of cortical circuits (Markram et al., 2015) would enable testing specific mechanistic hypotheses of functional hyperemia.

## Author contributions

All authors listed, have made substantial, direct and intellectual contribution to the work, and approved it for publication.

### Conflict of interest statement

The authors declare that the research was conducted in the absence of any commercial or financial relationships that could be construed as a potential conflict of interest. The reviewer DMT declared a shared affiliation, though no other collaboration, with one of the authors AD to the handling Editor, who ensured that the process nevertheless met the standards of a fair and objective review.
